# Multichamber Strain Imaging and Biomarker Profiling for 1-Year Risk Stratification in Pediatric Dilated Cardiomyopathy

**DOI:** 10.3390/life16030369

**Published:** 2026-02-24

**Authors:** Iolanda Muntean, Asmaa-Carla Hagau, Diana-Ramona Iurian, Beatrix Julia Hack, Diana Muntean, Horatiu Suciu

**Affiliations:** 1 Department of Paediatrics III, George Emil Palade University of Medicine, Pharmacy, Science, and Technology of Targu Mures, 540142 Targu Mures, Romania; iolanda.muntean@gmail.com; 2 Clinic of Pediatric Cardiology, Emergency Institute for Cardiovascular Diseases and Transplantation of Targu Mureș, 540139 Targu Mures, Romania; dianaiurian@yahoo.com (D.-R.I.); beatrix_hack@yahoo.com (B.J.H.); 3 Doctoral School of Medicine and Pharmacy, George Emil Palade University of Medicine, Pharmacy, Science, and Technology of Targu Mures, 540142 Targu Mures, Romania; 4 Pediatric Clinical Hospital, 550106 Sibiu, Romania; 5 Faculty of Medicine, George Emil Palade University of Medicine, Pharmacy, Science, and Technology of Targu Mures, 540142 Targu Mures, Romania; muntean.diana.24@stud.umfst.ro; 6 Department of Surgery IV, George Emil Palade University of Medicine, Pharmacy, Science, and Technology of Targu Mures, 540142 Targu Mures, Romania; horatiu.suciu@umfst.ro; 7 Clinic of Cardiovascular Surgery, Emergency Institute for Cardiovascular Diseases and Transplantation of Targu Mureș, 540139 Targu Mures, Romania

**Keywords:** pediatric cardiomyopathy, pediatric heart failure, speckle-tracking echocardiography, pediatric biomarkers

## Abstract

Dilated cardiomyopathy (DCM) in children is rare, but carries a high risk of progression to advanced heart failure (HF) and heart transplant (HTx). Improved short-term risk stratification is essential; however, robust pediatric prognostic tools remain limited. We aimed to evaluate the 1-year prognostic value of multichamber speckle-tracking echocardiography (STE) and biomarkers, including age-adjusted N-terminal pro-B-type natriuretic peptide (NT-proBNP) and vitamin D, in children with DCM. In this single-centre prospective cohort study, 29 children with idiopathic DCM and 27 age- and sex-matched healthy controls underwent standardised clinical, laboratory, and echocardiographic assessment. The primary endpoint was a 12-month composite of implantation of an implantable cardioverter-defibrillator (ICD), left-ventricular assist device (LVAD), HTx, or all-cause mortality. During a 1-year follow-up, 9/29 (31%) DCM patients experienced major events. Compared with event-free patients and controls, children with events had more impaired LVGLS (−5.99 ± 2.45% vs. −13.44 ± 6.88% and −19.98 ± 3.25%), lower LASr (10.97 ± 7.67% vs. 25.36 ± 10.28% and 44.0 ± 11.43%), and reduced RVFWSL (−15.32 ± 5.24% vs. −23.13 ± 8.55% and −24.78 ± 4.45%; all *p* < 0.01). Zlog NT-proBNP was markedly higher in the event group (5.37 [5.00–6.08] vs. 2.28 [0.71–3.68] and 0.14 [−0.02–0.88]). LVGLS, Zlog NT-proBNP, and LASr showed excellent discrimination for 1-year events (AUC 0.91, 0.91, and 0.87, respectively), with clinically applicable cut-offs (LVGLS ≥ −8%, Zlog NT-proBNP ≥ 4.6, LASr ≤ 21%). In conclusion, multichamber strain imaging combined with age-adjusted NT-proBNP provides clinically relevant, exploratory markers for short-term risk stratification in pediatric DCM, supporting earlier intensification of follow-up and timely referral for advanced heart failure therapies. These findings warrant validation in larger multicenter cohorts.

## 1. Introduction

Dilated cardiomyopathy is a rare but severe disease in the pediatric population, with an estimated annual incidence of 0.34–1.13 per 100,000 children and a substantial morbidity [1,2]. Despite advances in HF management, up to 30–40% of affected children will progress to advanced HF, die, or require HTx within five years of diagnosis [3].

Most therapeutic strategies and monitoring approaches continue to be extrapolated from adult HF guidelines, despite the substantial differences in pediatric versus adult DCM regarding aetiology, myocardial fibres and remodelling pathways [4,5]. In this context, reliable surrogate markers of adverse outcome are particularly valuable, as the low prevalence of pediatric DCM and the long follow-up required to capture mortality endpoints make randomised trials challenging. Because clinical deterioration often progresses insidiously, early identification of children at the highest risk is essential to guide monitoring, medical optimisation and timely referral for advanced therapy. Previous studies have highlighted several prognostic factors, including biomarkers such as NT-proBNP, echocardiographic indices of left ventricle (LV) systolic function, age at presentation or markers of functional capacity [6,7,8]. More recently, emerging evidence has also linked Vitamin D status with HF, suggesting that deficiency may contribute to adverse cardiac remodelling and worse clinical trajectories [9,10,11].

In the adult population, STE is firmly established in the management of cardiomyopathies, where strain indices have demonstrated prognostic value across different cardiomyopathy types [12,13,14]. Therefore, strain parameters were incorporated into screening and early diagnostic pathways. However, its application in pediatric practice remains comparatively limited because of the rarity of childhood cardiomyopathies and the challenges of conducting long-term outcome studies in this population. Consequently, the prognostic role of STE-derived parameters in children, particularly when integrated with clinical and biomarker information, is still insufficiently defined. Only a small number of pediatric studies have explored survival or major adverse outcomes in relation to strain measurements, and most have focused on single parameters rather than combined multimodal assessment [6,15,16]. In earlier work from our centre, we demonstrated that LVGLS is a sensitive indicator of myocardial impairment in pediatric DCM and correlates with disease severity [17]. Furthermore, we also showed the correlations between LASr, RVFWSL and disease severity [18,19].

This gap underscores the need for studies that evaluate strain-based markers within a comprehensive prognostic framework tailored to pediatric DCM. To our knowledge, no previous study in pediatric DCM has simultaneously assessed LV, right ventricle (RV), and LAS together with age-standardised NT-proBNP in relation to hard 1-year outcomes, nor has it shown clinically applicable cut-off values for risk stratification. By integrating multimodality deformation imaging with biomarker indices, our study aims to offer a more comprehensive prognostic framework than earlier single-parameter approaches. Additionally, the focus on a defined 12-month endpoint addresses the need for short- or mid-term risk stratification in the pediatric population, where timely escalation of care is crucial. Furthermore, Vitamin D has not previously been examined alongside strain parameters and NT-proBNP in pediatric DCM, adding a novel metabolic dimension to integrated risk stratification.

## 2. Materials and Methods

### 2.1. Study Design and Ethical Approval

This investigation represents a predefined sub-analysis of a prospective case–control research project conducted at the Pediatric Cardiology Clinic of the Emergency Institute for Cardiovascular Diseases and Transplantation, Targu Mures, Romania. The study protocol received approval from the Institutional Ethics Committee (Approval No. 566/2022, dated 20 January 2022) and complies with the Declaration of Helsinki.

This study uses the same pediatric DCM cohort previously described in our previous research. Still, it addresses a different research question, focusing exclusively on 1-year prognostic stratification based on echocardiographic deformation indices and biomarkers [17,18,19]. Also, due to the rarity of pediatric DCM and the limited number of outcome events, this study was designed as an exploratory, hypothesis-generating investigation aimed at identifying clinically relevant imaging and biomarker signals for short-term risk stratification.

No data, analyses, or text were duplicated from the previous publication. Before enrolment, all parents or legal guardians received written and verbal explanations of the study objectives and procedures. Written informed consent, authorising both participation and the anonymised use of imaging and laboratory data for scientific purposes, was obtained for every participant in accordance with institutional policy.

### 2.2. Study Population and Endpoints

This single-centre prospective cohort study included 29 children diagnosed with idiopathic DCM (DCM group) who were prospectively followed at our centre between January 2022 and December 2023. Inclusion criteria for DCM were: a confirmed diagnosis of idiopathic DCM based on the current guidelines (LV end-diastolic dimensions- LVEDD ˃ 2 z-scores and systolic dysfunction) associated with an age under 18 years [19]. Importantly, all consecutive patients meeting the diagnostic criteria during the study period were included, minimising selection bias and strengthening the validity of the cohort despite its limited size. Exclusion criteria encompassed congenital heart disease, structural valvular abnormalities, coronary anomalies, systemic hypertension, metabolic or neuromuscular disorders, secondary or infectious cardiomyopathies, patients with associated liver or kidney failure, and patients who received treatment with Vitamin D or any drug that is known to interfere with Vitamin D. None of the patients were excluded due to poor image quality or incomplete datasets.

For the current survival analysis, patients were divided into two groups according to the occurrence of major adverse events within 12 months: DCM without events (DCM−) and DCM with events (DCM+). The primary endpoint was 12-month major adverse cardiac events defined as: implantation of an ICD, LVAD, HTx or all-cause mortality. Time-to-event was calculated from the date of the baseline echocardiographic examination

A group of 27 healthy, age- and sex-matched controls was included for echocardiographic comparison of baseline function (Control Group). The children in the Control group were recruited from patients referred for innocent murmurs or sports clearance. All children underwent a normal cardiac evaluation, including a normal electrocardiogram and echocardiogram, with no known cardiovascular or systemic conditions.

### 2.3. Clinical and Laboratory Evaluation

Each participant underwent a standardised clinical assessment. Heart-failure severity was graded using the age-appropriate Ross classification (for <5 years) or NYHA functional class (≥5 years), according to the current guidelines [20]. Vital signs and anthropometric indices were recorded and converted to age-adjusted z-scores using CDC reference standards [21,22].

Venous blood sampling was performed during the same clinical encounter as the echocardiographic examination. Serum NT-proBNP was quantified using a chemiluminescent immunoassay platform (Roche Cobas, Mannheim, Germany). To account for the physiological influence of age and sex, NT-proBNP values were converted into standardised Z-scores, followed by log-transformation (Zlog NT-proBNP), using the validated reference calculator developed by the German Heart Centre Munich [23,24]. Serum 25-hydroxyvitamin D (25-OHD) was analysed by electrochemiluminescence immunoassay and was assessed at baseline. Vitamin D status was classified according to established thresholds: deficiency (<20 ng/mL), insufficiency (between 20 and 30 ng/mL), and sufficiency (˃30 ng/mL) [25].

### 2.4. Echocardiographic Acquisitions

A comprehensive transthoracic echocardiographic assessment was performed according to pediatric guidelines, by an experienced pediatric cardiologist, using the Phillips Epiq 7 ultrasound system equipped with 5–9 MHz transducers (Phillips Medical System, Bothell, WA, USA) [26]. All examinations were conducted without the need for sedation. Two-dimensional, M-mode, color Doppler and Tissue Doppler acquisitions were obtained from standard parasternal long- and short-axis views, as well as from apical four-, three- and two-chamber views (A4C, A3C, A2C). For each parameter, three consecutive cardiac cycles in sinus rhythm were averaged for the analysis.

Left ventricular end-diastolic diameter was measured in the parasternal long-axis view using M-mode at the level of the mitral valve leaflets and indexed to age and body size using established pediatric reference equations to derive LVEDD z-scores [27]. Left ventricular EF and LV shortening fraction (LVSF) were calculated using the biplane Simpson method. Mitral annular plane systolic excursion (MAPSE) was measured from the A4C view at the lateral mitral annulus. Tissue Doppler imaging was used to obtain peak systolic annular velocities (S′): lateral and medial S′ were recorded by placing a 2–3 mm sample volume at the corresponding mitral annular segments. Right-ventricular longitudinal function was evaluated by measuring tricuspid annular plane systolic excursion (TAPSE) in M-mode at the lateral tricuspid annulus. Right-ventricular systolic velocity (RV S′) was measured using tissue Doppler imaging at the lateral tricuspid annulus.

### 2.5. Speckle-Tracking Analysis

**Figure 1:**
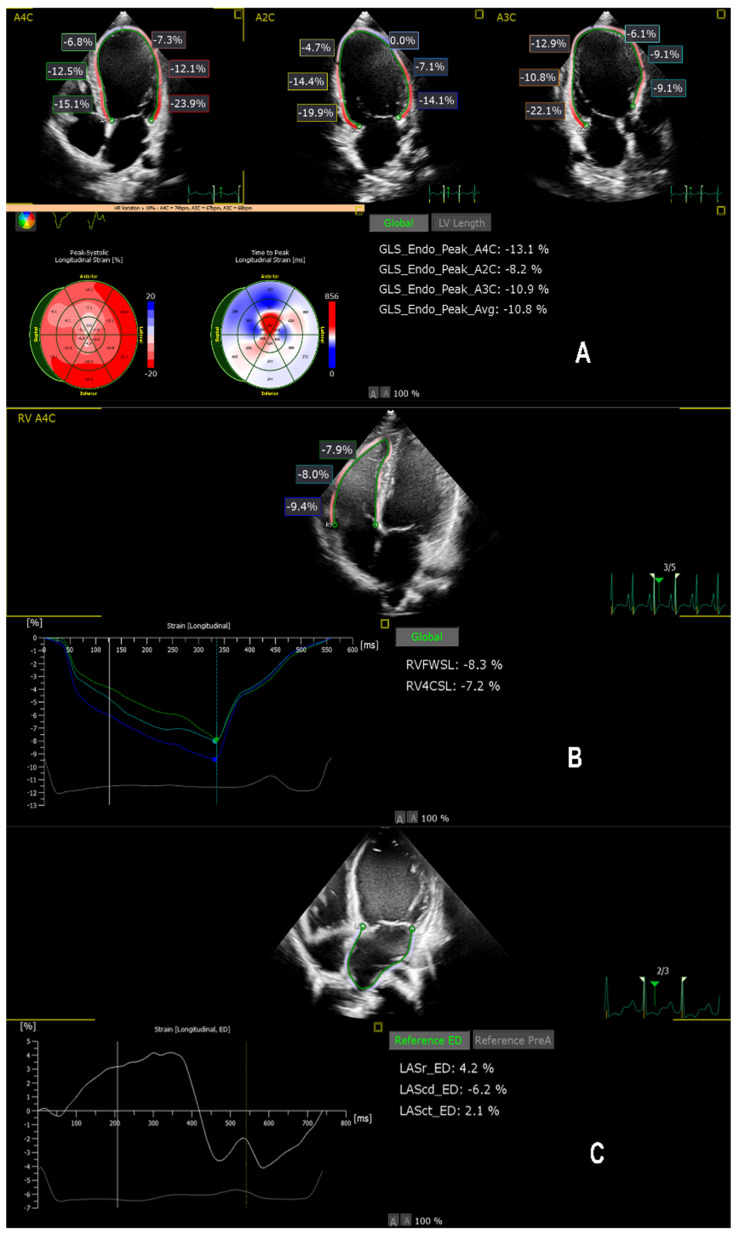
Representative multichamber speckle-tracking echocardiographic analysis in a patient with pediatric DCM. (**A**) Left ventricular global longitudinal strain (LVGLS) derived from standard apical views (A4C, A3C, A2C), illustrating reduced longitudinal deformation and heterogenous segmental strain distribution. Polar maps summarise regional peak systolic strain and time-to-peak strain. (**B**) Right ventricular free-wall longitudinal strain (RVFWSL) obtained from the focused A4C view, demonstrating impaired RV longitudinal deformation. (**C**) Left atrial strain analysis depicting reservoir (LASr), conduit and contractile phases, reflecting altered atrial mechanics. In all panels, colored curves represent segmental strain profiles over the cardiac cycle, while numerical values correspond to peak systolic strain measurements automatically computed by the software.

Myocardial deformation analysis was performed offline using a dedicated vendor-independent platform (QLAB, version 15.5, Phillips Medical Systems, Bothell, WA, USA) equipped with the AutoStrain modules. All images were acquired at frame rates between 60 and 90 frames/second to ensure optimal quality. The cine-loops were exported in DICOM format and analyzed offline by a single experienced operator blinded to clinical data and outcomes (Figure 1).

Left-ventricular longitudinal strain was assessed from apical views, using an 18-segment model. Endocardial contours were generated automatically and manually adjusted when required. Global LV longitudinal strain (LVGLS) was calculated as the average peak systolic longitudinal strain of all analyzable segments, using an 18-segment model. For regional assessment, segments were grouped into three anatomical levels (basal, mid-ventricular, and apical). Peak systolic strain values within each level were averaged to obtain basal, mid-ventricular, and apical longitudinal strain indices. Strain values are reported as percentages, with more negative values indicating better systolic shortening.

Mitral annular displacement (TMAD) was assessed as an additional marker of LV longitudinal mechanics using the AutoStrain-TMAD tool in the A4C view. The software automatically identified the mitral annular plane and tracked the motion of three points (medial, septal, and mid-annular) toward the LV apex during systole. TMAD medial, TMAD septal, and TMAD midpoint (mm and %) were exported for statistical analysis.

Right-ventricular deformation was evaluated with the AutoStrain-RV module using RV-focused A4C views [28]. Right-ventricular free-wall longitudinal strain (RVFWSL) was evaluated using the RV-focused A4C view. The endocardial border of the RV was traced semi-automatically, and tracking was inspected frame-by-frame. The three free-wall segments (basal, mid-ventricular, and apical) were included for quantification, and RVFWSL was calculated as the mean peak systolic longitudinal strain of these segments.

Left-atrial deformation was analysed using the AutoStrain-LA module from the A4C view. The LA endocardial border was traced manually at ventricular end-systole, excluding the pulmonary veins and left-atrial appendage. The software then tracked the atrial wall throughout the cardiac cycle. The zero-strain reference frame was set at LV end-diastole. Left-atrial reservoir strain (LASr) was defined as the peak positive strain during ventricular systole, representing the atrial reservoir phase and reflecting atrial compliance and left-sided filling pressures.

Sector width and imaging depth were adjusted to optimise temporal resolution while preserving full myocardial visualisation. For each view, three consecutive sinus beats were selected for analysis, and strain values were averaged across these beats to reduce beat-to-beat variability. When multiple acquisitions were available, the loop with the best image quality and most stable tracking was chosen.

Although similar acquisition and analysis protocols were used as in our previous work, the present study differs in its prognostic focus, multichamber integration, and event-based outcomes.

(A) Apical chamber-views from two-dimensional echocardiography with endocardial tracking and the corresponding left ventricular global longitudinal strain (LVGLS) bull’s-eye plot, demonstrating severely reduced longitudinal deformation.
(B) Right-ventricular-focused apical four-chamber view with right-ventricular free wall longitudinal strain (RVFWSL).
(C) Apical four-chamber view with left atrial strain curves illustrating impaired reservoir function.

### 2.6. Statistical Analysis

Continuous variables were tested for normality using the Shapiro–Wilk test and presented as mean ± standard deviation (SD) or median [interquartile range, IQR]. Categorical variables are reported as counts and percentages. For continuous variables, overall group differences were explored using one-way ANOVA or Kruskal–Wallis tests, as appropriate, followed by pairwise comparisons. Comparisons between groups were performed using independent samples *t*-tests for normally distributed continuous variables and Mann–Whitney U tests for skewed data. Categorical variables were compared using the χ^2^ test or Fisher’s exact test, as appropriate. Given the exploratory design, *p*-values were not adjusted for multiple comparisons.

Correlations between biomarkers and echocardiographic parameters were assessed using Pearson’s correlation coefficients for normally distributed variables and Spearman’s rank correlation for non-normal variables.

Segmental LV longitudinal strain was analyzed using a linear mixed-effects model, with segment and clinical group entered as fixed effects and subject as a random effect. Main effects and segment × group interaction terms were evaluated to assess global and regional patterns of dysfunction.

Receiver operating characteristic (ROC) curves were constructed to evaluate the ability of echocardiographic and biomarker variables to predict 12-month major adverse events. The area under the ROC curve (AUC) with 95% confidence intervals (CI) was calculated for each parameter. Optimal prognostic cut-off values for continuous predictors were derived using Youden’s index. Event-free survival was estimated using the Kaplan–Meier method. Univariable Cox proportional hazards regression models were used to explore the association between continuous predictors and the composite endpoint. Given the limited number of events, multivariable Cox models were not constructed to avoid model overfitting and unstable hazard ratio estimates. Also, because of the rarity of pediatric DCM and the exploratory nature of the study, no a priori sample size or power calculation was performed. All tests were two-sided, and a *p*-value < 0.05 was considered statistically significant. Statistical analyses were performed using IBM SPSS Statistics (version 26, IBM Corp., Armonk, NY, USA) and GraphPad Prism (version 10, GraphPad Software, San Diego, CA, USA).

## 3. Results

### 3.1. Baseline Demographic, Anthropometric, Hemodynamic, and Biomarker Characteristics

Compared with controls, children with DCM were younger, had lower body mass index (BMI), and lower systolic and diastolic blood pressures (BP). Also, Zlog NT-proBNP was markedly elevated in both DCM subgroups, with the highest levels observed in the DCM+ group. Vitamin D concentrations were lower in DCM than in controls.

Within the DCM cohort, functional status differed markedly. Among the DCM group, most were classified as NYHA/Ross Class I–II (65%). Patients in the DCM+ group were exclusively in advanced functional classes: 45% in class III (n = 4) and 55% in class IV (n = 5). No significant differences were observed in anthropometric measurements, BP, body mass index (BMI), or body surface area (BSA), indicating that functional status and biomarkers, but not somatic growth, distinguished patients with adverse outcomes (Table 1).

### 3.2. Conventional Echocardiographic Measurements

Conventional echocardiographic indices demonstrated marked systolic impairment in children with DCM compared with healthy controls (Table 2). Left-ventricular end-diastolic dimensions and z-scores were significantly increased in both DCM subgroups, indicating advanced LV chamber dilation. Left ventricular systolic function was substantially reduced, with a stepwise decline in LVEF and LVSF from controls to DCM− and most markedly in DCM+.

Longitudinal annular function was also impaired, with significantly lower MAPSE and reduced lateral and medial S′ velocities in both DCM groups. Right ventricular systolic function showed a similar pattern of deterioration: TAPSE and RV S′ were significantly reduced in patients with events, indicating biventricular involvement in advanced disease. Overall, the conventional echocardiographic profile confirms progressive LV dilation and global systolic dysfunction across DCM subgroups, with more pronounced abnormalities in patients who developed major adverse events.

### 3.3. Ventricular and Atrial Deformation Indices

Left ventricular deformation indices showed a clear and progressive deterioration across groups (Table 3). Left ventricular GLS was markedly reduced in the DCM cohort compared to controls (*p* < 0.01 for all pairwise comparisons). A similar graded impairment was observed across all ventricular levels: basal, mid-ventricular, and apical strain values were significantly decreased in both DCM subgroups, with the most pronounced dysfunction consistently present in the DCM+ group (all *p* < 0.01).

Mitral annular displacement was also significantly reduced in DCM, reflecting impaired longitudinal mechanics. Medial, septal, and mid-point TMAD values were lower in both DCM subgroups compared with controls (all *p* < 0.01). Differences between DCM− and DCM+ patients trended in the expected direction, but did not reach statistical significance, suggesting that TMAD captures global systolic impairment but offers limited discriminative ability for early event prediction.

Right ventricular free-wall longitudinal strain demonstrated a distinct pattern: values in DCM− were comparable to controls, whereas patients with events showed a marked reduction (*p* < 0.01 vs. both controls and DCM−), indicating selective RV involvement in advanced disease.

Left atrial reservoir strain showed the strongest chamber-specific separation after LVGLS. LASr was significantly reduced in DCM− (25.36 ± 10.28% vs. 44 ± 11.43% in controls, *p* < 0.01) and profoundly impaired in DCM with events (10.97 ± 7.67%, *p* < 0.01). LASr differences were highly significant between all groups (all *p* < 0.01), highlighting its sensitivity to elevated filling pressures and advanced diastolic dysfunction.

Collectively, these deformation indices demonstrate a coherent pattern of multi-chamber mechanical impairment in pediatric DCM. LVGLS, RVFWSL, and LASr showed the strongest discrimination between patients with and without subsequent adverse events.

### 3.4. Correlations Between Biomarkers and Echocardiographic Parameters

Vitamin D levels showed a moderate, statistically significant positive correlation with LVEF (r = 0.43, *p* < 0.05). In addition, 25-OHD demonstrated borderline associations with RVFWSL and LASr (*p* between 0.05–0.07), suggesting a potential link between reduced vitamin D levels and both RV dysfunction and impaired atrial compliance. No significant correlations were observed between vitamin D and LVGLS.

Zlog NT-proBNP showed strong linear associations with several echocardiographic parameters. Higher NT-proBNP correlated very strongly with impaired LVGLS (r = 0.84, *p* < 0.01) and moderately to strongly with reduced LVEF (r = −0.71, *p* < 0.01). Significant correlations were also observed with RVFWSL (r = 0.58, *p* < 0.01) and LASr (r = −0.53, *p* < 0.01). Overall, Zlog NT-proBNP showed the strongest association with LVGLS (R^2^ = 0.70), indicating that strain impairment closely reflects neurohormonal activation and hemodynamic burden in pediatric DCM.

### 3.5. Segmental Left Ventricular Longitudinal Strain Patterns

**Figure 2:**
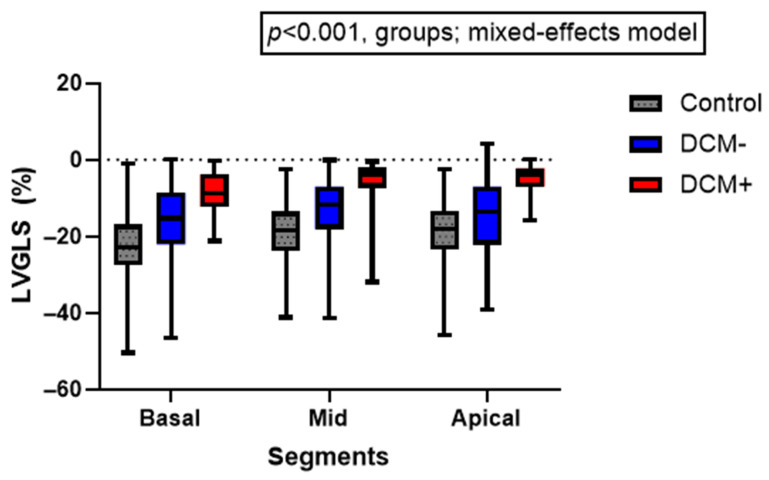
Segmental LV longitudinal strain (basal, mid-ventricular, apical) in controls, DCM− and DCM+. Box-and-whisker plots illustrate progressively impaired strain across all ventricular levels in DCM, with the most profound dysfunction observed in the DCM+ group. A mixed-effects model demonstrated a significant overall group effect (*p* < 0.001), indicating a consistent and parallel reduction in segmental strain from controls to DCM− and DCM+. Apical strain showed the greatest separation between subgroups, reflecting more advanced myocardial remodelling in patients who developed major adverse events.

Segmental LVGLS differed significantly across ventricular levels and across clinical groups (both *p* < 0.01). In contrast, the segment × group interaction was not significant (*p* > 0.05), indicating a parallel pattern of impairment across the basal, mid, and apical segments (Figure 2). Post hoc analysis revealed progressively reduced strain from controls to DCM patients without events, with the most pronounced impairment observed in those with events.

Comparing the two DCM subgroups directly (DCM− vs. DCM+), the largest difference in mean strain values was observed at the apical level (Δ9.69%), exceeding the basal (Δ5.65%) and mid-ventricular (Δ7.99%) (Table 4). These findings demonstrate uniform, but progressively worsening, segmental dysfunction across all ventricular levels. Apical GLS showed the largest separation between DCM subgroups, suggesting advanced remodelling and higher clinical risk in patients who developed major adverse events.

### 3.6. Prognostic Performance of Imaging and Biomarkers (ROC Analysis)

**Figure 3:**
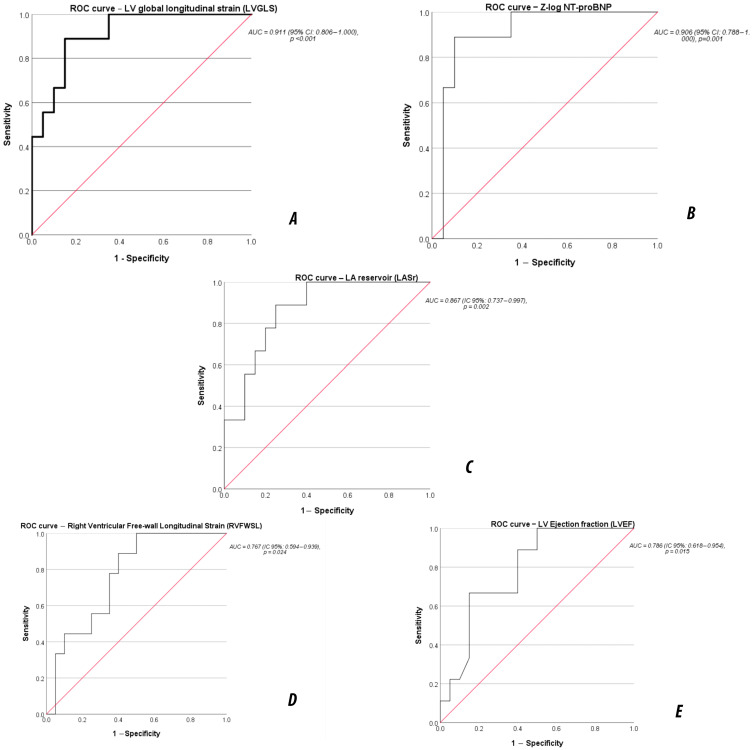
Receiver-operating characteristic (ROC) curves for predicting 1-year major adverse events in pediatric DCM. (**A**) Left ventricular global longitudinal strain (LVGLS) demonstrated excellent discriminative ability (AUC 0.911, 95% CI 0.806–1.000, *p* < 0.001). (**B**) Zlog NT-proBNP showed similarly high prognostic performance (AUC 0.906, 95% CI 0.788–1.000, *p* = 0.001). (**C**) Left atrial reservoir strain (LASr) provided strong predictive value (AUC 0.867, 95% CI 0.737–0.997, *p* = 0.002). (**D**) Right ventricular free-wall longitudinal strain (RVFWSL) showed moderate discriminatory capacity (AUC 0.767, 95% CI 0.594–0.939, *p* = 0.024). (**E**) Left ventricular ejection fraction (LVEF) demonstrated modest performance (AUC 0.786, 95% CI 0.618–0.954, *p* = 0.015). Cut-off values derived from Youden’s index: LVGLS ≤ −8%, Zlog NT-proBNP ≥ 4.6, LASr ≤ 21%, LVEF ≤ 35%, RVFWSL ≥ −20%.

Receiver-operating characteristic analysis identified LVGLS, Zlog NT-proBNP, and LASr as the strongest predictors of 12-month adverse outcomes (Table 5, Figure 3). LVGLS demonstrated excellent discrimination, with an optimal cut-off of ≥−8%, followed by Zlog NT-proBNP at a threshold ≥4.6. LASr also provided excellent prognostic separation, with a cut-off of ≤21%. LVEF and RVFWSL demonstrated moderate discriminative performance, with clinically relevant thresholds of ≤35% and ≥−20%, respectively.

The identified cut-off values should be interpreted as exploratory thresholds derived from this cohort and are intended to inform hypothesis generation, rather than define clinical decision limits.

### 3.7. Kaplan–Meier Event-Free Survival

**Figure 4:**
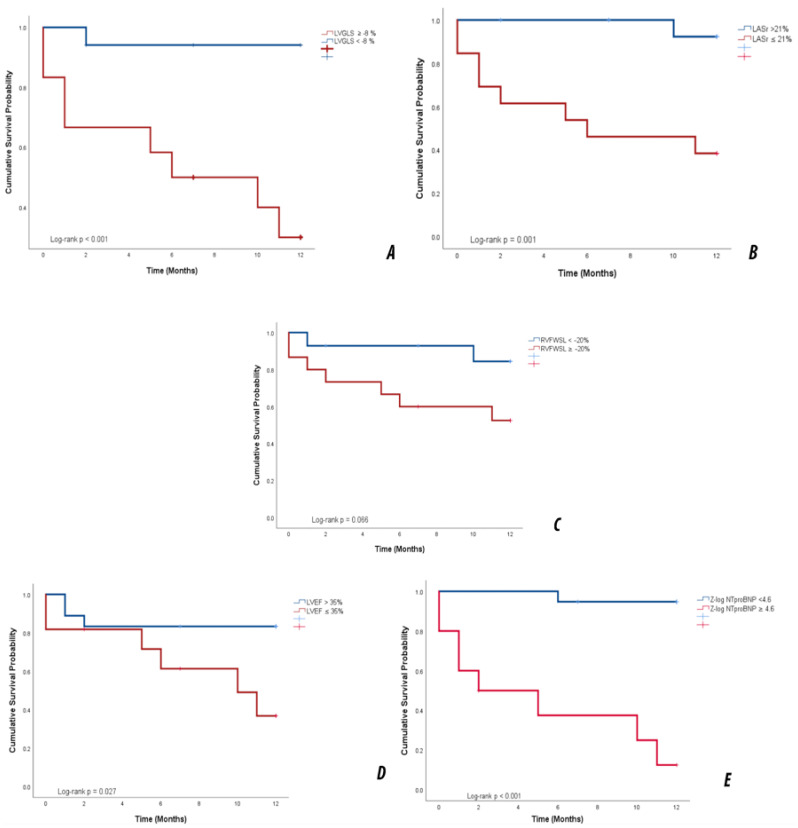
Kaplan–Meier event-free survival (**A**) Patients with LVGLS < −8% experienced significantly reduced event-free survival. (**B**) LASr ≤ 21% was associated with markedly shorter event-free survival (**C**). RVFWSL ≥ −20% showed a trend toward poorer survival (**D**) LVEF ≤35% identified patients at higher risk of events (**E**) Elevated Z-log NT-proBNP (≥4.6) was associated with the worst prognosis.

Event-free survival (EFS) was evaluated for the composite endpoint of ICD implantation, LVAD implantation, heart transplantation, or death. Among the 29 patients included, 9 experienced a major event (31%), while 20 (69%) were censored. The cumulative EFS estimates were 83% at 5 months, 79% at 7 months, 72% at 10 months and approximately 67% at 12 months. These data indicate that around two-thirds of patients remained free of major adverse events at 1 year (Figure 4).

Kaplan–Meier curves demonstrated distinct prognostic profiles for all echocardiographic and biomarker-derived predictors used for risk stratification in pediatric DCM.

Patients with impaired LVGLS (>−8%) had substantially worse outcomes (66.7% vs. 5.9% event rate). Median EFS was 6 months in the impaired group, whereas the preserved-strain group did not reach the median. Mean survival was 6.78 ± 1.43 versus 11.41 ± 0.57 months. The difference between curves was highly significant (log-rank χ^2^ = 12.654, *p* < 0.001).

Patients with elevated biomarker levels (Zlog NT-proBNP ≥ 4.6) exhibited the poorest prognosis (80% events) with a median survival of 2 months, while the low-risk group (5.3% events) did not reach the median. Mean survival differed substantially (5.15 ± 1.54 vs. 11.68 ± 0.31 months, χ^2^ = 23.007, *p* < 0.001).

Left Atrial Reservoir Strain ≤21% was associated with a 61.5% event rate, compared with 6.2% in the preserved-strain group. The impaired group reached a median survival of 6 months, while patients with LASr >21% did not reach the median. Mean survival was 6.61 ± 1.42 vs. 11.85 ± 0.15 months (χ^2^ = 10.364, *p* = 0.001).

A threshold of LVEF ≤ 35% yielded a moderate prognostic value. Event rates were 54.5% versus 16.7%, with a median survival of 10 months in the impaired group. The difference between curves reached significance (χ^2^ = 4.882, *p* = 0.027), though with a smaller effect size than strain-based parameters.

Patients with RVFWSL ≥ −20% had a higher event rate (46.7% vs. 14.3%), and lower mean survival (8.06 ± 1.27 vs. 11.05 ± 0.76 months). Although the curves diverged, significance was not reached (χ^2^ = 3.374, *p* = 0.066), indicating a slight trend toward prognostic relevance.

## 4. Discussion

This present study builds upon a series of previous investigations from our group that characterised LV, atrial and RV deformation patterns in pediatric DCM [17,18,19]. Those studies primarily focused on cross-sectional functional characterisation, chamber-specific mechanics and correlations with disease severity at a single time point. However, the current study addresses a distinct and complementary research question by prospectively evaluating the short-term prognostic value of multichamber strain parameters integrated with biomarker profiles. Specifically, this work is the first from our cohort to simultaneously link LVGLS, LASr, and RVFWSL to hard 12-month clinical outcomes. In addition, we provide event-based analysis, ROC-derived exploratory thresholds and Kaplan–Meier survival curves, shifting the focus from functional description to clinical risk stratification. Even though the cohort overlaps with prior publications, the analytical framework, endpoints and clinical interpretation presented here are fundamentally different. Despite the exploratory nature, our findings aimed to emphasise the central involvement of myocardial deformation and neurohormonal activation in disease progression and short-term prognosis among children with DCM, rather than myocardial mechanics alone.

### 4.1. Prognostic Value of Strain-Derived Parameters

In our study, LVGLS was the strongest mechanical predictor of adverse outcomes, consistent with its sensitivity to early systolic impairment and its established role in reflecting myocardial fibrosis and remodelling. Less negative LVGLS values were strongly associated with major adverse events. These findings align with prior pediatric and adult studies demonstrating that deterioration in LVGLS precedes the decline in conventional systolic indices and correlates better with disease severity and clinical outcome [6,7]. In a multicenter study, de Boer et al. confirmed that impaired LVGLS is an independent predictor of death or HTx in pediatric DCM patients during follow-up, whereas traditional LV systolic markers such as LVEF, LVSF and even circumferential strain did not retain prognostic significance [6]. The prognostic value of LVGLS is further supported by findings in adults. Raafs et al. showed that LVGLS remained independently associated with a composite of sudden death, life-threatening arrhythmias and HF hospitalisation [29]. In our cohort, LVGLS ≥ −8% had the highest predictive accuracy (AUC 0.91), and the patients in the high-risk LVGLS subgroups had markedly reduced event-free survival.

Importantly, regional strain analysis revealed a diffuse reduction in longitudinal deformation across all myocardial levels compared with healthy controls. In our cohort, basal strain was markedly decreased in both DCM subgroups, with a similar stepwise deterioration observed in the mid-ventricular region and in the apical segments. These findings indicate a loss of the physiological base-to-apex gradient and are consistent with previous pediatric DCM studies reporting global and diffuse impairment of longitudinal myocardial deformation [6,17]. The significant separation of mean strain values between DCM patients with or without events, particularly at the apical level, suggests that apical deformation impairment may signal advanced myocardial remodelling and increased risk.

Left atrial strain reservoir also demonstrated significant prognostic value. Reduced LASr reflects elevated left-sided filling pressures and impaired atrial compliance, both markers of advanced diastolic dysfunction. In the present cohort, LASr ≤ 21% discriminated event-free survival and demonstrated an AUC of 0.87. These findings are consistent with observations by Wozniak et al., who reported that children with DCM who experienced adverse outcomes exhibited markedly impaired atrial mechanics, including significantly lower LASr [16].

Right-ventricular free-wall strain demonstrated intermediate prognostic performance. Although statistical significance was borderline, patients with RVFWSL ≥ −20% had lower event-free survival, suggesting that early RV dysfunction may accompany or follow left-sided decompensation. Right ventricular impairment is increasingly recognised as a determinant of outcomes in pediatric cardiomyopathy [15]. Prior work supports this observation: Al-Biltagi et al. showed that children with DCM exhibit significant reductions in RV longitudinal systolic velocities and deformation indices, with RV strain correlating strongly with HF severity and LV systolic dysfunction [30]. Similarly, Groner et al. demonstrated that impaired RV function independently predicts adverse outcomes in idiopathic pediatric DCM, highlighting RV involvement as a key contributor to clinical deterioration [31]. Collectively, these data reinforce the notion that RV dysfunction is not merely a late marker of biventricular failure but an integral component of disease progression in pediatric DCM. It is important to mention that, despite the fact that these cut-off values offer clinically thresholds for short-term risk stratification, their prognostic accuracy should be interpreted cautiously, given the exploratory nature of the analysis.

### 4.2. Biomarkers Role and Their Integration with Strain Imaging

The prognostic role of NT-proBNP in our cohort is consistent with observations from larger prospective pediatric registries. Schmitt et al. showed that NT-proBNP concentrations bear a strong, dose-dependent relationship to the risk of death or HTx in pediatric patients with HF, with an increased risk in patients who exhibited persistent biomarker elevation. Furthermore, they reported that the association between NT-proBNP and clinical endpoints was remarkably similar across different cardiac pathologies and comparable to adult data, suggesting that myocardial stretch, rather than the specific disease substrate, drives the risk [8]. Also, the study published by Van Der Meulen et al. demonstrated that both baseline values and dynamic measurements of NT-proBNP are powerful determinants of survival [7]. In contrast, Kim et al. reported that the initial NT-proBNP value did not predict clinical outcomes in pediatric DCM patients, likely due to the variability of baseline NT-proBNP, which is influenced by the initial LV dysfunction or hemodynamic presentation. Instead, they showed that NT-proBNP level at 3 months may be a better long-term prognostic [32]. Our findings extend these observations by showing that, even as a single baseline measurement, Zlog NT-proBNP provides excellent discrimination of event-risk (AUC 0.91). Using an age-adjusted, log-transformed metric like Zlog NT-proBNP rather than raw NT-proBNP values, we reduced the physiological age-dependent variability, well known in the pediatric population. Furthermore, whereas Van Der Meulen et al. focused on serial biomarker and dimensional changes during disease progression, our study adds advanced deformation imaging with biomarker-based risk assessment. The strong association between Zlog NT-proBNP and LVGLS underscores the close coupling between neurohormonal activation and intrinsic myocardial dysfunction in pediatric DCM. Additional correlations with RVFWSL and LASr indicate that RV and atrial function also deteriorate in parallel with rising ventricular pressures and wall stress.

In the present cohort, lower 25-OHD concentrations were modestly associated with impaired systolic function, as reflected by a positive correlation with LVEF, and showed borderline associations with RVFWSL and LASr. However, no significant association was observed between vitamin D and LVGLS in our analysis. In contrast, a recent study evaluating myocardial deformation in adults without overt cardiovascular disease demonstrated a clear association between vitamin D status and subclinical systolic dysfunction [33]. Further supporting the link between vitamin D status and myocardial mechanics, a prospective interventional study in adults without overt cardiovascular disease demonstrated that vitamin D deficiency is associated with impaired LV deformation and increased epicardial fat thickness [34]. In the pediatric population, the studies are very limited. A recent study of patients with β-thalassemia major, vitamin D deficiency and insufficiency was associated with adverse echocardiographic markers, including larger LV end-diastolic dimensions, altered diastolic function and a strong positive correlation between vitamin D levels and LVGLS [35]. Additionally, in infants with hypocalcemia and concomitant Vitamin D deficiency exhibited more marked LV strain impairment, and both global longitudinal and circumferential strains improved significantly after correction of hypocalcemia and vitamin D status [36]. These findings suggest that vitamin D status may be indirectly associated with cardiac performance, acting as a potential modulator of hemodynamic load and ventriculo-atrial interaction, rather than a direct determinant of intrinsic myocardial deformation. In our cohort, vitamin D levels were not significantly associated with LVGLS, which is considered a relatively load-dependent marker of intrinsic myocardial contractile function. In contrast, Vitamin D showed modest associations with more load-dependent parameters, such as LVEF, RVFWSL, and LASr. This pattern supports the interpretation that vitamin D may influence cardiovascular status predominantly through hemodynamic pathways rather than through direct effects on myocardial deformation. Therefore, the absence of an association between Vitamin D and LVGLS does not contradict prior studies; actually, it suggests that Vitamin D may act as a secondary modulator of global performance rather than a primary determinant of longitudinal systolic deformation. However, given the small sample size and the single time-point measurement, the prognostic implications remain exploratory. Also, it should be noted that the biomarker assessment was limited to baseline measurements; longitudinal trajectories of NT-proBNP or vitamin D levels may have additional prognostic insights beyond those captured in this analysis.

Overall, the strengths of this study include its prospective design, comprehensive multichamber strain assessment using vendor-independent software, and integration of deformation imaging with biomarker profiling in a pediatric cohort with DCM. At the same time, the exploratory nature of this analysis and the limited sample size due to rare pediatric diseases warrant cautious interpretation. Rather than refining clinical decision thresholds, these findings are intended to create more hypotheses and support the design of larger, multicenter studies in order to validate short-term risk stratification.

### 4.3. Limitations

This study has several limitations inherent to its design. First, the sample size was small, reflecting the low prevalence of paediatric DCM and the single-centre nature of the cohort. Second, due to the limited number of clinical events, we did not perform multivariable Cox regression modelling to avoid model instability. However, this approach aligns with methodological standards in rare paediatric disorders, where univariable analyses and survival curves provide more reliable and interpretable prognostic information. Despite this limitation, the consistency of findings across strain indices, biomarkers, ROC analysis, and Kaplan–Meier curves enhances the internal validity of the results. However, prognostic cut-off values derived from univariable ROC analyses may overestimate predictive performance in small datasets with a limited number of events; the thresholds should be interpreted as hypothesis-generating and require validation in larger studies. Third, echocardiographic strain measurements may be affected by image quality and vendor-dependent variability. All speckle-tracking analyses were performed using uniform acquisition protocols by the same experienced operator, which ensured internal consistency. However, formal assessment of intra- and interobserver variability was not performed and should be considered a methodological limitation of the present study [37]. Fourth, NT-proBNP values can be influenced by age-related physiological variation. However, the Zlog transformation mitigates this limitation but does not eliminate it. Finally, as an observational study, causality cannot be established, and unmeasured confounders may still influence the results. These findings should therefore be viewed as exploratory and hypothesis-generating. Larger, prospective multicentre studies are needed to confirm these observations and to establish robust, standardised prognostic thresholds for paediatric DCM.

## 5. Conclusions

Pediatric DCM is characterized by heterogeneous progression and high early morbidity. This study demonstrates that multimodality imaging combined with age-adjusted biomarker profiling may enhance early risk stratification in pediatric DCM. Each parameter provides complementary prognostic information, in addition to conventional echocardiographic indices alone. While prospective multicenter studies are needed to validate these findings, our results support the potential role of multichamber strain imaging and biomarkers as exploratory tools for early risk stratification in pediatric DCM.

## Figures and Tables

**Table 1:** Baseline characteristics of controls and pediatric DCM patients.

	Controls (n = 27)	DCM− (n = 20)	DCM+ (n = 9)	*p*-Value		
				(C vs. DCM−)	(C vs. DCM+)	(DCM− vs. DCM+)
Age(years)	15.4 [11.6–16.7]	10 [2.3–16.8]	16 [13–16]	0.06	0.8	0.2
Male	−50% (n = 13)	−80% (n = 16)	−55% (n = 5)			
Female	−50% (n = 13)	−20% (n = 4)	−45% (n = 4)			
BMI (kg/m^2^)	20.3 ± 3	17.2 ± 3.7	20.5 ± 5.4	0.01	0.86	0.06
BSA (m^2^)	1.57 ± 0.28	1.31 ± 0.53	1.32 ± 0.49	0.06	0.13	0.95
sBP (mmHg)	107.7 ± 9.59	99.95 ± 9.91	106.6 ± 14.69	0.01	0.79	0.12
25-OHD (ng/mL)	31.14 ± 8.67	20.92 ± 8.8	17.03 ± 4.56	<0.01	<0.01	0.4
Zlog NT-proBNP	0.14 [−0.88 to −0.02]	2.28 [0.71–3.68]	5.37 [5–6.08]	<0.01	<0.01	0.06

Data are mean ± SD or median [IQR]. Comparisons between groups were performed using independent *t*-tests or Mann–Whitney U tests, as appropriate. Categorical variables are shown as counts and percentages. Abbreviations: DCM—dilated cardiomyopathy; DCM− = DCM without major events; DCM+—DCM with major events; BMI—body mass index; BSA—body surface area; sBP—systolic blood pressure; Zlog NT-proBNP—age-adjusted z-scored logaritmic N-terminal pro-B-type natriuretic peptide. 25-OHD—25-dihydroxy-Vitamin D.

**Table 2:** Conventional echocardiographic parameters of controls and pediatric DCM patients.

	Controls (n = 27)	DCM− (n = 20)	DCM+ (n = 9)	*p*-Value		
				(C vs. DCM−)	(C vs. DCM+)	(DCM− vs. DCM+)
MAPSE (cm)	1.73 ± 0.26	1.08 ± 0.36	0.89 ± 0.16	<0.01	<0.01	0.26
S′ lateral (cm/s)	11.08 ± 2.62	7.93 ± 2.23	6.4 ± 1.55	<0.01	<0.01	0.1
S′ medial (cm/s)	8.7 [8.16–9.7]	6.38 [5.16–7.56]	4.64 [4.13–6.2]	<0.01	<0.01	0.35
LVEDD z-score	0.6 [−0.4 to −1.7]	2.87 [2.18–3.78]	3.47 [2.97–4.15]	<0.01	<0.01	0.1
LVEF (%)	72 ± 4.78	48.21 ± 17.33	30.12 ± 12.43	<0.01	<0.01	<0.01
LVSF (%)	41.13 ± 4.23	25.58 ± 10.04	15.82 ± 5.14	<0.01	<0.01	<0.01
TAPSE (cm)	2.29 ± 0.47	2.08 ± 0.37	1.69 ± 0.6	0.3	<0.01	0.1
RV S′ (cm/s)	13.15 ± 1.42	12.82 ± 2.98	9.62 ± 1.77	0.8	<0.01	<0.01

Data are mean ± SD or median [IQR]. Abbreviations: DCM—dilated cardiomyopathy; DCM− = DCM without major events; DCM+—DCM with major events; MAPSE—mitral annular plane systolic excursion; S′—peak systolic annular velocity; LVEDD—LV end-diastolic diameter; LVEF—LV ejection fraction; LVSF—LV shortening fraction; TAPSE—tricuspid annular plane systolic excursion; RV S′—RV systolic velocity.

**Table 3:** Left ventricular, right ventricular and atrial deformation parameters in controls and pediatric DCM patients.

	Control (n = 27)	DCM− (n = 20)	DCM+ (n = 9)	*p*-Value		
				(C vs. DCM−)	C vs. DCM+)	(DCM− vs. DCM+)
LVGLS (%)	−19.98 ± 3.25	−13.44 ± 6.88	−5.99 ± 2.45	<0.01	<0.01	<0.01
LV basal (%)	−22.16 [−39 to −13.58]	−15.23 [−22.09 to −8.71]	−8.77 [−12.8 to −3.8]	<0.01	<0.01	<0.01
LV mid-ventricular (%)	−18.41 [−23.86 to −13.55]	−11.8 [−18.1 to −7]	−3.85 [−7.35 to −1.97]	<0.01	<0.01	<0.01
LV apical (%)	−18.04 [−23.41 to −13.44]	−13.58 [−22.16 to −7.05]	−3.46 [−7.04 to −2.02]	<0.01	<0.01	<0.01
TMAD midpoint (mm)	12.2 [10.9–13.65]	7.05 [5.32–9.2]	5.6 [3.55–7.9]	<0.01	<0.01	0.22
TMAD midpoint (%)	16.2 [14.28–18.23]	11.25 [7.27–13.53]	7.2 [3.95–9.2]	<0.01	<0.01	0.07
RVFWSL (%)	−24.78 ± 4.45	−23.13 ± 8.55	−15.32 ± 5.24	0.54	<0.01	<0.05
LASr (%)	44 ± 11.43	25.36 ± 10.28	10.97 ± 7.67	<0.01	<0.01	<0.01

Data are mean ± SD or median [IQR]. Strain values are reported as absolute percentages (with more negative values indicating better systolic deformation). DCM—dilated cardiomyopathy; DCM− = DCM without major events; DCM+ = DCM with major events; LVGLS—LV global longitudinal strain; TMAD—mitral annular displacement; RVFWSL—RV free-wall longitudinal strain; LASr—LA reservoir strain.

**Table 4:** Differences in regional LVGLS between study groups.

Group Comparison	LV Basal Δ(%)	LV Mid-Ventricular Δ(%)	LV Apical Δ(%)
Controls vs. DCM−	5.88%	6.07%	4.03%
Controls vs. DCM+	11.98%	11.79%	11.16%
DCM− vs. DCM+	5.65%	7.99%	9.69%

Values are absolute mean GLS differences (Δ) by LV level. Abbreviations: DCM− = dilated cardiomyopathy without events; DCM+ = dilated cardiomyopathy with events; GLS—global longitudinal strain.

**Table 5:** ROC analysis for prediction of 1-year major adverse events in pediatric dilated cardiomyopathy.

Parameter	AUC (95%CI)	*p*-Value	Optimal Cut-off	Sensitivity (%)	Specificity (%)
LVGLS (%)	0.911 (0.806–1.000)	<0.001	≥−8%	88.9	85.0
Zlog NT-proBNP	0.906 (0.788–1.000)	0.001	≥4.6	88.9	90.0
LASr (%)	0.867 (0.737–0.997)	0.002	≤21%	88.9	75.0
LVEF (%)	0.786 (0.618–0.954)	0.015	≤35%	66.7	85.0
RVFWSL (%)	0.767 (0.594–0.939)	0.024	≥−20%	88.9	60.0

AUC, *p*-values, and optimal cut-off values derived from Youden’s index. Sensitivity and specificity refer to the ability of each marker to discriminate between patients with and without 1-year events. Abbreviations: LVGLS—LV global longitudinal strain; Zlog NT-proBNP—age-adjusted z-scored logaritmic N-terminal pro-B-type natriuretic peptide; LASr—LA reservoir strain; LVEF—LV ejection fraction; RVFWSL—RV free-wall longitudinal strain.

## Data Availability

The data underlying this article cannot be shared publicly due to the sensitive nature of pediatric clinical information and the risk of re-identification. The data will be shared upon reasonable request to the corresponding author.

## Abbreviations

The following abbreviations are used in this manuscript:

DCM	Dilated cardiomyopathy
HF	Heart failure
HTx	Heart transplant
STE	Speckle-tracking echocardiography
NT-proBNP	N-terminal pro-B-type natriuretic peptide
ICD	Implantable cardioverter-defibrillator
LVAD	Left-ventricular assist device
LVGLS	Left ventricular global longitudinal strain
LASr	Left atrial strain reservoir phase
RVFWSL	Right-ventricular free-wall longitudinal strain
LV	Left ventricle
RV	Right ventricle
DCM−	Dilated cardiomyopathy group without events
DCM+	Dilated cardiomyopathy group with events
25-OHD	25-hydroxyvitamin D
Zlog NT-proBNP	age-adjusted z-scored logarithmic N-terminal pro-B-type natriuretic peptide
LVEF	Left ventricle ejection fraction
LVSF	Left ventricle shortening fraction
MAPSE	Mitral annular plane systolic excursion
S′	Peak systolic annular velocities
TAPSE	Tricuspid annular plane systolic excursion
A2C/A3C/A4C	Apical two-, three-, four-chamber view
RV S′	Right ventricular systolic velocity
TMAD	Mitral annular displacement
ROC	Receiver operating characteristic
AUC	Area under the curve
BMI	Body mass index
BP	Blood pressure
BSA	Body Surface Area
EFS	Event-free survival
